# Enhancing Early Detection of Necrotizing Soft Tissue Infections: The Role of the Laboratory Risk Indicator for Necrotizing Fasciitis (LRINEC) Score

**DOI:** 10.7759/cureus.61620

**Published:** 2024-06-03

**Authors:** Satya S Bhat, Pallavi H. R., Shanmukha Koppolu, Mohammed M Ahmed, Aparnna U Nair, Madhusudhanan D., Naveenkumar Nallathambi, Yogesh S., Prashanth A., Roshan Prasad, Gaurav Mittal

**Affiliations:** 1 Internal Medicine, Cardiff and Vale University Health Board, Cardiff, GBR; 2 General Surgery, Adichunchanagiri Institute of Medical Sciences, B.G. Nagara, IND; 3 Urology, Queen Alexandra Hospital, Portsmouth, GBR; 4 Internal Medicine, Altnagelvin Area Hospital, Londonderry, GBR; 5 General Surgery, Hindu Mission Hospital, Chennai, IND; 6 Internal Medicine, Madras Medical College, Chennai, IND; 7 Internal Medicine, Rajiv Gandhi Government General Hospital, Chennai, IND; 8 Physiology, Mahatma Gandhi Institute of Medical Sciences, Wardha, IND; 9 Medicine and Surgery, Jawaharlal Nehru Medical College, Datta Meghe Institute of Higher Education and Research, Wardha, IND; 10 Internal Medicine, Mahatma Gandhi Institute of Medical Sciences, Wardha, IND; 11 Research and Development, Student Network Organization, Mumbai, IND

**Keywords:** assessment, mortality, early diagnosis, necrotizing fasciitis, lrinec, infections, skin disease

## Abstract

Background

There is great variation in the etiology, predisposing organisms, incidence, clinical characteristics, severity, and consequences of skin and/or subcutaneous tissue infections. Extensive necrosis of the subcutaneous tissues and fascia is a characteristic of necrotizing soft tissue infections, which are frequently deadly. To change the course of treatment, this study highlights the need to find a tool that can quickly and accurately identify patients with necrotizing fasciitis (NF) and assist in making an early treatment decision.

Methodology

A prospective evaluation of 30 individuals with soft tissue infections was conducted using the laboratory risk indicator for necrotizing fasciitis (LRINEC). The patients were classified as low, intermediate, and high risk for the start of NF based on their LRINEC score. To assess the importance of the LRINEC score in forecasting the start of NF and its clinical consequences, patients in each group underwent appropriate management and statistical analysis.

Results

This study included 28 males (93.3%) and two females (6.7%). The associated p-value, recorded as 0.039, signifies statistical significance in the observed area under the receiver operating characteristic (ROC) curve. The p-value in risk categorization was found to be 0.296, which suggests that LRINEC helps in risk categorization with 100% sensitivity when used as a screening tool.

Conclusion

The early detection of necrotizing soft tissue infections, such as NF, is vital. The LRINEC score, based on routine lab tests, accurately distinguishes these infections. With high sensitivity and significant p-values, it helps stratify patients, guiding timely interventions and saving lives.

## Introduction

A rapidly spreading inflammatory fascia infection, necrotizing fasciitis (NF) results in subcutaneous tissue necrosis as a byproduct. The thickness of the subcutaneous layer directly relates to the rate of spread. Along the fascial plane, NF advances. The incidence of NF ranges from 0.4 to 7.7 per 100,000 [1]. Several studies have shown a rise in incidence over the past few decades [2].

Types of soft-tissue necrotizing infection can be divided into four classes according to the types of bacteria infecting the soft tissue. This classification is discussed in detail by Paz Maya et al. in their study on NF [3].

A serious infection of the subcutaneous soft tissues, mostly the superficial and frequently the deep fascia, is called NF. It is often an acute condition; however, it can sometimes proceed in a sub-acute manner. NF can infect any area of the body; however, it typically affects the extremities, particularly the legs. The abdominal wall, the perianal and groin regions, and surgical incisions are other sites of predilection. Abrasions, lacerations, insect bites, burns, laparotomies performed in the presence of peritoneal soiling (such as in cases of penetrating abdominal trauma or perforated viscus), surgical procedures (such as hemorrhoidectomy or vasectomy), perirectal abscesses, decubitus ulcers, and intestinal perforations are the most common sources of infection gateways. A foreign object, such as a chicken bone or toothpick, a rectosigmoid tumor, or hidden diverticulitis, might be the cause of intestinal perforation. NF originating from these intestinal origins might manifest in the lower limbs, groin, or abdominal wall. By extension along the psoas muscle, infection from intestinal sources can reach the lower extremities, and through a colo-cutaneous fistula, it can reach the abdominal wall. NF, in particular, can arise in the context of parenteral drug addiction, diabetes mellitus, and alcoholism. There are several terms used to describe this condition, namely progressive synergistic infection gangrene, NF, Fournier gangrene, cancrum oris, and Meleney's gangrene, all of which are types of flesh-eating bacterial diseases [4].

Recognition of the characteristic features and the rapidly progressive clinical course of the disease aids in the diagnosis of NF [5]. Olafsson et al. [6] demonstrate that the trademark manifestation of NF is extreme pain and tenderness over the involved skin and underlying muscle.

The cost and accessibility of computed tomography, magnetic resonance imaging, and frozen section biopsy restrict their routine use in the assessment of soft tissue infections.

Hence, Wong et al. [7] designed a simple scoring system, the laboratory risk indicator for necrotizing fasciitis (LRINEC), which is based on routine laboratory investigations that are readily available at most centers, and that can help distinguish NF from other soft tissue infections. The six laboratory variables that are measured at the time of presentation - hemoglobin, total leukocyte count, serum glucose, serum sodium, serum creatinine, and serum C-reactive protein - are used to construct the LRINEC score.

Although Wong et al. [7] suggested the LRINEC score is capable of detecting even clinically early cases of NF, other studies [8-11] have not yielded satisfactory results so far to validate the LRINEC score for routine use.

Therefore, the purpose of this study is to evaluate the use and efficacy of the LRINEC score as a diagnostic tool for NF in the early stages of the disease.

## Materials and methods

Study setting and design

This prospective observational study commenced after obtaining ethical clearance from the Institutional Ethical Committee of the Adichunchanagiri Institute of Medical Sciences, B.G. Nagara, India (AIMS/IEC/2124/2019-20), along with written informed consent from the patients. The study included 30 patients aged 15 to 75 years who were diagnosed with NF in the surgical wards of Adichunchanagiri Hospital and Research Centre, affiliated with Adichunchanagiri Institute of Medical Sciences.

Selection criteria

The inclusion criteria for this study comprised patients aged 18 to 75 years who presented with severe soft tissue infections. This age range was selected to ensure a broad representation of adult patients who are most likely to experience such infections. The severity of the soft tissue infection was a key factor in inclusion, focusing on those cases that required more intensive evaluation and treatment. The exclusion criteria were designed to eliminate variables that could confound the study results. Patients who required multiple admissions solely due to soft tissue infection were excluded, as their recurrent nature could indicate underlying conditions not representative of the general population with severe soft tissue infection cases. Additionally, patients with surgical site infections were excluded to focus the study on infections not related to recent surgical interventions, which could have different etiologies and treatment responses. Finally, patients who had received antibiotic treatment within the last 48 hours or at least three doses prior to presentation were excluded to avoid the potential bias that pre-treatment could introduce in the assessment of infection severity and response to treatment. This criterion ensured that the study population consisted of patients whose infection status had not been recently altered by antibiotic therapy, allowing for a clearer evaluation of the natural progression and initial presentation of severe soft tissue infections.

Data sources and variables

Clinical examinations were conducted on patients who exhibited symptoms indicative of soft tissue infections. To gather comprehensive data, a semi-structured observational checklist, which had been pre-evaluated for reliability, was utilized to collect demographic and covariate information related to the infections. Each participant was assessed using the LRINEC scoring system, which helped in quantifying the severity of their condition. Following this, all collected variables were analyzed statistically to determine the significance of the LRINEC score in predicting clinical outcomes.

Routine investigations performed on the patients included a series of standard tests to assess various health parameters. These tests encompassed hemoglobin levels, total leukocyte count, differential leukocyte count, erythrocyte sedimentation rate (ESR), platelet count, blood grouping and Rh typing, blood urea, serum creatinine, fasting blood sugar, postprandial blood sugar, antibiotic culture and sensitivity tests, serum electrolytes, and C-reactive protein levels. Additionally, X-rays and ultrasonography of the affected limb were conducted as necessary to provide further diagnostic insights.

Special investigations were also carried out, which involved obtaining tissue samples for culture and sensitivity testing to identify the causative organisms and their antibiotic resistance patterns. Tissue fluid was also collected for gram staining to provide a rapid preliminary identification of bacterial pathogens. It is important to note that no animal experiments were involved in this study, ensuring that all procedures were ethically conducted on human subjects only.

Statistical analysis

Data was input into an Excel workbook (Microsoft® Corp., Redmond, WA, USA). The means and standard deviations of the quantitative variables as well as the frequencies and proportions of the qualitative variables were computed as descriptive statistics. Analysis of variance (ANOVA) examined group mean differences. A receiver operating characteristic (ROC) curve identified the best LRINEC score cutoff value, with sensitivity and specificity. The significance level was set at 5%. IBM SPSS Statistics for Windows, Version 20 (Released 2011; IBM Corp., Armonk, New York, United States) was used for analysis.

## Results

Table 1 shows the age distribution of the 30 study subjects. There were five subjects aged 30-39, six aged 40-49, 11 aged 50-59, six aged 60-69, one aged 70-79, and one aged 80-89. The highest number of subjects was in the 50-59 age group.

**Table 1:** Age-wise distribution

Age	Number of subjects	Percentage (%)
30-39	5	16.67%
40-49	6	20%
50-59	11	36.67%
60-69	6	20%
70-79	1	3.33%
80-89	1	3.33%
Total	30	100%

Table 2 displays the sex-wise distribution of the study subjects. Out of 30 subjects, two were female (6.7%) and 28 were male (93.3%), making a total of 100%.

**Table 2:** Sex-wise distribution

Gender	Frequency	Percentage (%)
Female	2	6.7%
Male	28	93.3%
Total	30	100.0%

Table 3 presents the LRINEC score criteria.

**Table 3:** Laboratory risk indicator for necrotizing fasciitis (LRINEC) score mg/L: milligrams per liter; mm^3^: cubic millimeter; gm/dL: grams per deciliter; mmol/L: millimoles per liter

Variable	Score
C-reactive protein (mg/L)	
<150	0
≥150	4
Total white cell count (per mm^3^)	
<15	0
15-25	1
>25	2
Hemoglobin (gm/dL)	
>13.5	0
11-13.5	1
<11	2
Sodium (mmol/L)	
≥135	0

Table 4 presents the risk categorization of the study subjects. Out of 30 subjects, three (10.0%) were classified as high risk, two (6.7%) as intermediate risk, and 25 (83.3%) as low risk. This distribution shows that the majority of the subjects fell into the low-risk category.

**Table 4:** Risk categorization

Risk category	Frequency	Percentage (%)
High	3	10.0%
Intermediate	2	6.7%
Low	25	83.3%
Total	30	100.0%

Table 5 categorizes 30 patients by LRINEC risk: 25 low-risk, two intermediate-risk, and three high-risk. Erythema, pain, and swelling were present in all patients. Bullae occurred in 20 patients, necrosis in 11, and fever in 14. Crepitus was absent in all. Tachycardia was seen in 10 patients, and hypotension in four patients (none in the low-risk group).

**Table 5:** Clinical presentation in necrotizing fasciitis LRINEC: laboratory risk indicator for necrotizing fasciitis

LRINEC category	Low risk	Intermediate risk	High risk
Total patients	25	2	3
Erthyema	25	2	3
Pain	25	2	3
Swelling	25	2	3
Bullae	17	1	2
Necrosis	8	1	2
Crepitus	0	0	0
Fever	9	2	3
Tachycardia	5	2	3
Hypotension	0	2	2

**Figure 1:**
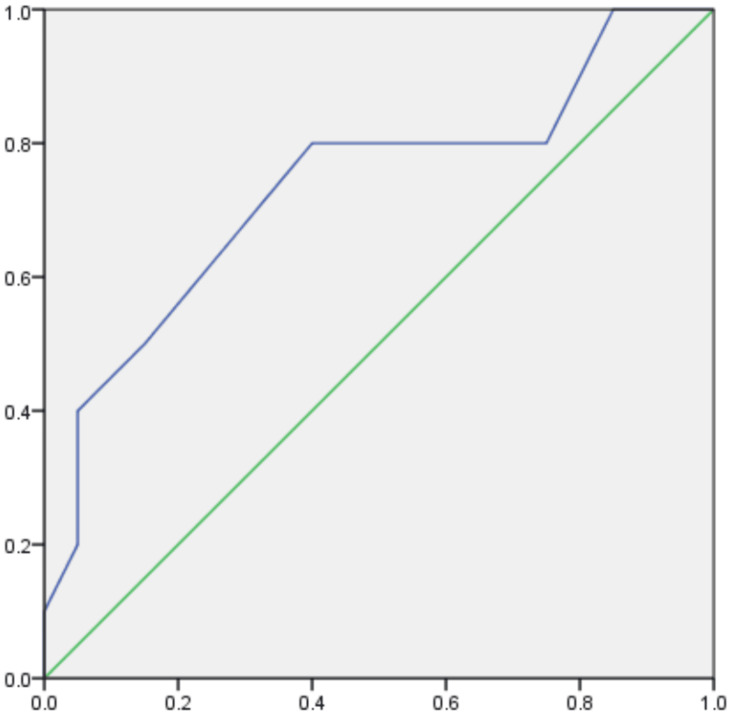
ROC curve ROC: receiver operating characteristic curve The X-axis represents specificity, while the Y-axis represents sensitivity The green line represents the ideal curve, while the blue line represents the outcome curve of this study

Table 6 presents the area under the ROC curve for the LRINEC score, a diagnostic tool utilized in identifying necrotizing soft tissue infections. The area under the ROC curve, indicative of the score's ability to differentiate between patients with and without these infections, is reported as 0.735. Accompanying this value, the standard error is listed as 0.105, suggesting some variability in the estimate. The associated p-value, recorded as 0.039, signifies statistical significance in the observed area under the ROC curve. Figure 1 represents the ROC curve.

**Table 6:** Area under ROC curve ROC: receiver operating characteristic curve; LRINEC: laboratory risk indicator for necrotizing fasciitis p-value is considered to be statistically significant at <0.05

LRINEC score			Asymptotic 95% CI	
					Area	Std. error	p-value	Lower bound	Upper bound
0.735	0.105	0.039	0.53	0.94

Table 7 outlines the LRINEC score thresholds and their associated sensitivity and specificity for identifying necrotizing soft tissue infections. Sensitivity starts high at 100% for a score of 0.00, while specificity is 0%. Sensitivity remains relatively high across increasing scores, but specificity also rises, indicating a trade-off between correctly identifying positives and negatives.

**Table 7:** Coordinates of the curve LRINEC: laboratory risk indicator for necrotizing fasciitis

LRINEC score		
Positive if greater than or equal to	Sensitivity	Specificity
0.00	100	0
1.50	100	15
2.50	80	25
3.50	80	60
4.50	50	85
6.00	40	95
7.50	20	95
10.00	10	100
13.00	0	100

Table 8 presents p-values for risk categorization using the LRINEC score. Three risk categories are identified: low, intermediate, and high. Each category's mean LRINEC score, standard deviation, and 95% confidence interval for the mean are provided. The analysis indicates variability in LRINEC scores across risk categories, with associated p-values indicating the statistical significance of these differences.

**Table 8:** p-value in risk categorization using LRINEC score LRINEC: laboratory risk indicator for necrotizing fasciitis p-value is considered to be statistically significant at <0.05

Risk category	N	Mean ± SD	95% confidence interval for mean		F-value	p-value
			Lower bound	Upper bound	1.273	0.296
Low	25	51.28 ± 10.546	46.93	55.63		
Intermediate	2	52.5 ± 3.536	20.73	84.27		
High	3	62.33 ± 19.858	13	111.66		
Total	30	52.47 ± 11.443	48.19	56.74		

## Discussion

Necrotizing soft tissue infections are deadly infectious diseases that proceed fatally. They are most common in obese diabetic patients and injectable drug users, and they can have a wide range of clinical manifestations that are linked to severe sepsis. Changes in the biochemical markers are anticipated in the context of sepsis due to the related systemic inflammatory response syndrome.

These alterations are measured by the LRINEC score, which also indicates if NF will be present. Seldom do other soft tissue infections, such as cellulitis and abscesses, result in an inflammatory state severe enough to alter the laboratory variables in this way.

Patients with NF often have a delayed clinical onset, which might result in incorrect diagnosis. Localized pain, swelling, and erythema are the most frequent symptoms; nevertheless, it is uncommon for all three to appear at the same time. Since NF patients may not always present with a typical clinical appearance, laboratory testing can be helpful in both diagnosing the condition and determining its severity. The LRINEC can be used to assess the severity of NF in general.

This prospective study of 30 patients with soft tissue infections included 28 males (93.3%) and two females (6.7%). In our study, the majority of the subjects were males with 93.3%, whereas it was 51%, 73.10%, and 73.3% in Faucher et al. [12], Chowdary et al. [13], and Pratheek et al. [14], respectively.

The most common age group was between 50-59 years accounting for 36.6% of cases followed by between 40-49 years and 60-69 years, with each group bearing 20% of cases. The mean age group was 52.46. As compared to the study conducted by Pratheek et al. [14], the common age groups were 41-50 years and 61-70 years, both accounting for 30% of cases each. The second group is 51-60 years.

The majority of the patients presented with multiple symptoms. The most common manifestations at presentation were erythema, swelling, and pain, observed in almost all subjects in our study. Other symptoms included bullae (56.6%), fever (30%), necrosis (26.6%), tachycardia (16.6%), and hypotension (13.3%). In contrast, Kumar et al. [15] reported fever (86%), tenderness (78%), erythema (73%), necrosis (61%), tachycardia (53%), and bullae (38%). A study conducted by Wong et al. [16] showed erythema (100%), pain (98%), bullae (45%), necrosis (14%), fever (53%), and hypotension (18%). Pain and swelling were the main presenting symptoms of NF in the study conducted by Soitkar et al. [17]. Fever was the next most common symptom observed in 90% of the patients. Blister formation and cuticular necrosis were observed in 29.5% of the patients.

With a p-value of 0.039, the LRINEC scoring system demonstrates higher sensitivity in identifying the onset of NF in soft tissue infections. Therefore, it can be considered an excellent screening tool for early diagnosis and intervention, thereby potentially reducing morbidity and mortality in patients with NF.

Limitations of the study

The small sample size of 30 patients limits the generalizability of the findings, and the predominance of male participants (93.3%) may skew the results, not providing a balanced view of how NF affects females. Additionally, the age distribution, with the majority in the 50- to 59-year age group, might not represent the broader population affected by NF. The study focused on patients with severe soft tissue infections, excluding milder cases which might present differently, limiting the applicability of the results to all soft tissue infections. The reliance on clinical and laboratory findings without incorporating advanced imaging techniques further limits comprehensive assessment.

## Conclusions

The prompt identification and management of necrotizing soft tissue infections, such as NF, are critical for patient outcomes due to the potentially life-threatening nature of these conditions. The LRINEC score, derived from routine laboratory investigations, serves as a valuable tool in distinguishing NF from other soft tissue infections. With its enhanced sensitivity and significant p-values, the LRINEC scoring system enables clinicians to effectively stratify patients with severe soft tissue infections, facilitating timely interventions and ultimately improving patient well-being. Early recognition and risk stratification through the LRINEC score are pivotal steps in ensuring prompt and appropriate management, ultimately leading to better outcomes for individuals affected by necrotizing soft tissue infections.
